# Supplementary material for: Single-Molecule Characterization of the Interactions between Extracellular Chaperones and Toxic α-Synuclein Oligomers
Source: Cell Rep. 2018 Jun 19;23(12):3492–500. doi: 10.1016/j.celrep.2018.05.074 (PMC6024880; doi:10.1016/j.celrep.2018.05.074)
Supplement: Document S1. Figures S1–S3 [file mmc1.pdf]

Cell Reports, Volume *23*

Supplemental Information

Single-Molecule Characterization of the Interactions between Extracellular Chaperones and Toxic α-Synuclein Oligomers

Daniel R. Whiten, Dezerae Cox, Mathew H. Horrocks, Christopher G. Taylor, Suman De, Patrick Flagmeier, Laura Tosatto, Janet R. Kumita, Heath Ecroyd, Christopher M. Dobson, David Klenerman, and Mark R. Wilson

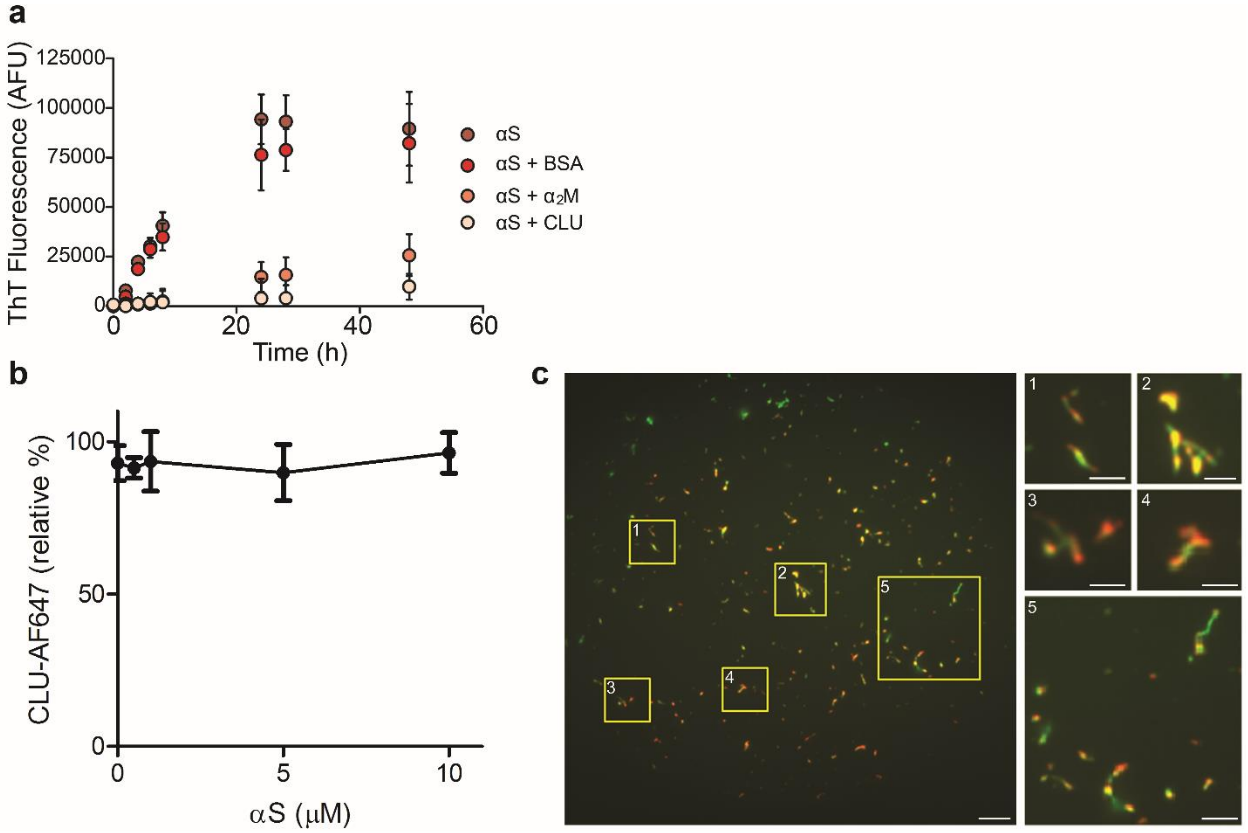

Figure S1. *Related to Figures 1 and 2.* **(a)** αS (70 μM) was incubated alone or with BSA (as a negative control), α_2M or CLU (each at 0.7 μM) for 48 h at 37 °C and 200 rpm shaking**.** The time points were taken during the reaction and the extent of aggregation was quantified by ThT (20 μM) fluorescence using a Clariostar platereader (BMG LabTech; excitation 440-15 nm, emission 500-25 nm). Data shown are means ± SD of three replicate treatments. **(b)** CLU-AF647 (100 nM) was incubated with shaking for 1 h with various concentrations of αS fibrils (equivalent monomer concentrations are shown, diluted from a 70 μM aggregation reaction incubated for 48 h). The absence of significant quenching was observed when the total fluorescence intensity of the solution was measured and found to be independent from the number of fibrils present. **(c)** TIRF microscopy was used to show that CLU can indeed bind to fibrils. Fibrils were identified using thioflavin T and are shown in green, CLU-AF647 is shown in red, coincidence appears yellow. Panels at right show larger versions of areas indicated by numbered yellow boxes. The scale bars are 2 μm for the main panel, and 1 μm for each of the smaller panels.

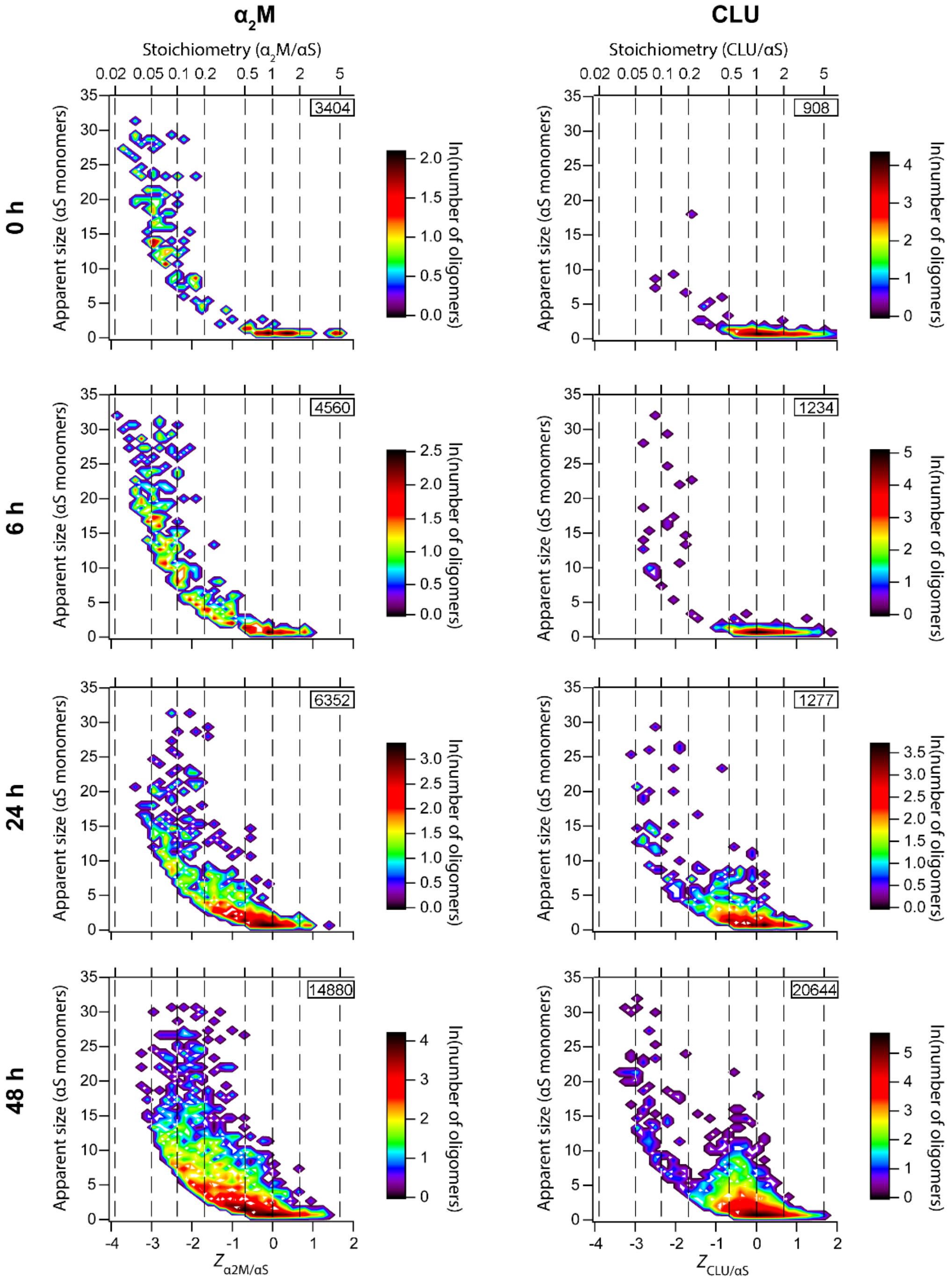

Figure S2. *Related to Figures 1 and 2.* αS^{A90C}-AF488 (70 µM) and either α_2M-AF647 (0.7 µM; left column) or CLU-AF647 (0.7 µM; right column) were co-incubated at 37 °C, with shaking at 200 rpm, in PBS (pH 7.4). Samples were taken from the aggregation reaction at the indicated times and the formation of αS-chaperone complexes was quantified by single-molecule TCCD. $Z_{chaperone/\alpha S}$ represents the logarithm of the apparent ratio of chaperone to client in each oligomer. The numbers in the inserts indicate the number of complexes represented in the plot. Data shown are representative of at least three separate experiments.

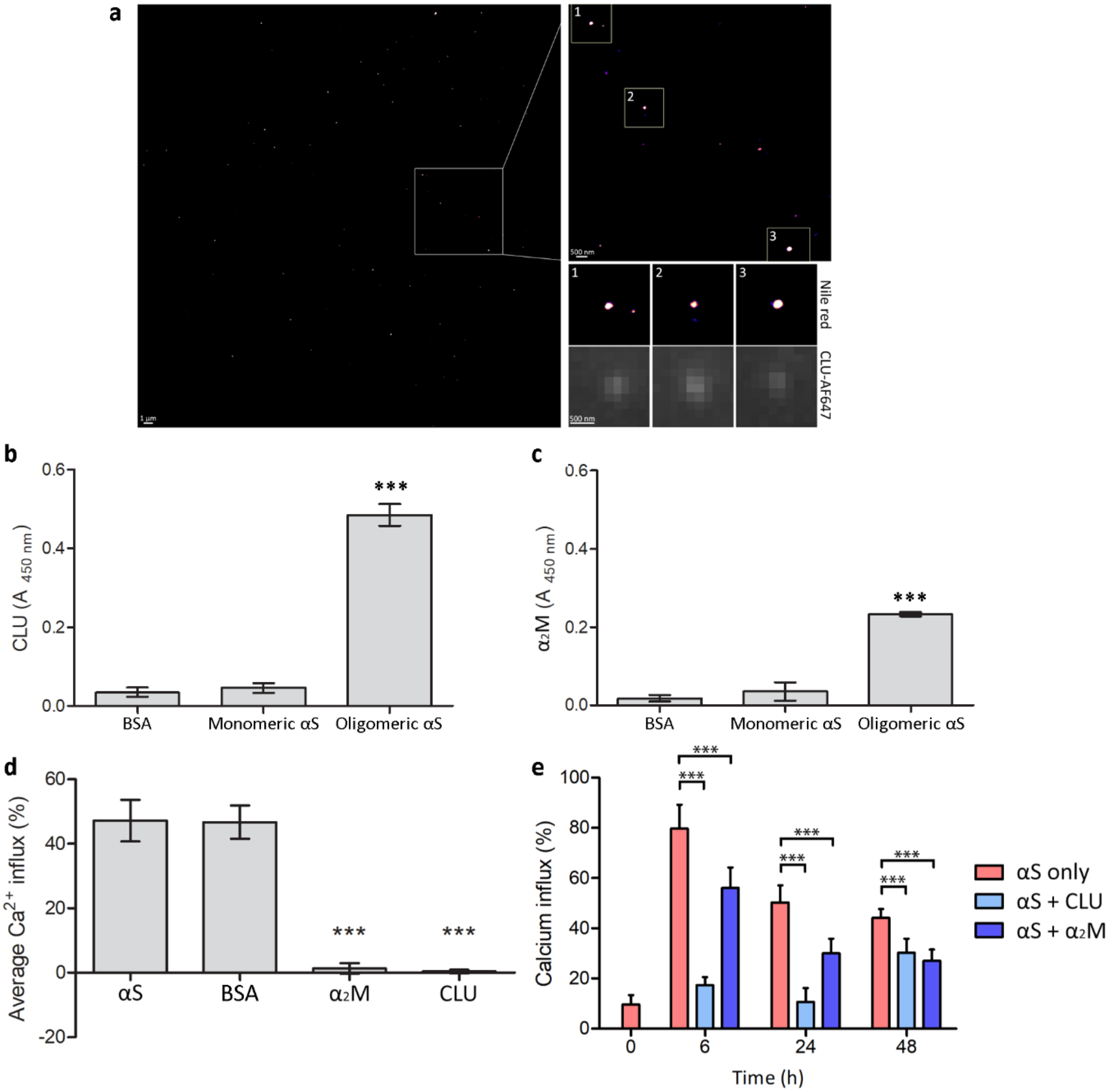

Figure S3. *Related to Figure 3.* **(a)** Monomeric αS was incubated at a concentration of 70 μM under aggregating conditions for 9 h. Aliquots were taken from the aggregation reaction and diluted to a concentration of 3 μM in PBS with 5 nM Nile red for super-resolution imaging. Small aggregates consistent with the expected appearance of oligomers were observed (see the three zoomed panels labelled 'Nile red'). These aggregates were preincubated with CLU-AF647 for 5 min before imaging; panels labelled 'CLU-AF647' are images of the same region as the super-resolution Nile red image but show the diffraction-limited fluorescence of colocalised CLU-AF647. **(b and c)** BSA, monomeric αS or αS incubated under aggregating conditions for 9 h (oligomeric αS) were adsorbed to a microplate. After BSA blocking and incubation with the indicated chaperone the quantity of bound (b) CLU or (c) α_2M was measured by ELISA. *** $p < 0.001$; values shown are means ± SD of three independent experiments. **(d)** αS was incubated under aggregating conditions for 9 h. Aliquots were removed from the aggregation reaction and samples of αS were diluted and added to Cal-520-containing vesicles after preincubation in the presence or absence of BSA, α_2M or CLU at equimolar concentrations to αS (50 nM). Unlike α_2M and CLU, BSA did not counteract the membrane permeability induced by αS. Data shown are means ± SD of 9 fields of view (at least 1000 vesicles); *** $p < 0.001$. **(e)** *CLU and α_2M reduce αS-induced membrane disruption.* α_2M or CLU (700 nM) was incubated with αS (70 μM) for 48 h under aggregating conditions. At the time-points taken both α_2M and CLU decreased the vesicle permeabilisation caused by αS aggregates. Data shown are means ± SD of 16 fields of view (at least 800 vesicles); *** $p < 0.001$.
