# Supplementary material for: Single-Molecule Characterization of the Interactions between Extracellular Chaperones and Toxic α-Synuclein Oligomers
Source: Cell Rep. 2018 Jun 19;23(12):3492–500. doi: 10.1016/j.celrep.2018.05.074 (PMC6024880; doi:10.1016/j.celrep.2018.05.074)
Supplement: Document S2. Article plus Supplemental Information [file mmc2.pdf]

Cell Reports

Article

Single-Molecule Characterization of the Interactions between Extracellular Chaperones and Toxic α-Synuclein Oligomers

Graphical Abstract

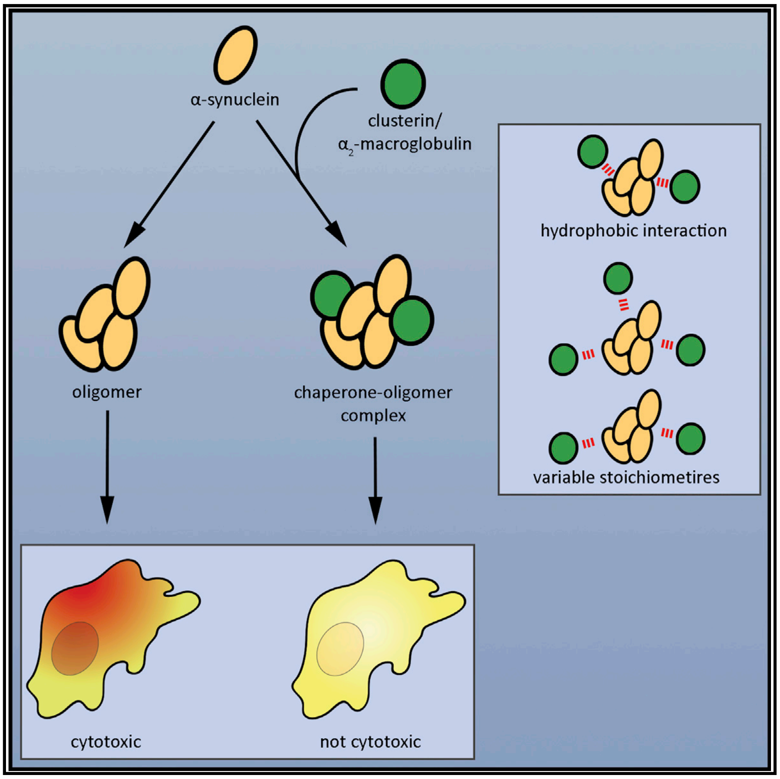

Highlights

- Two extracellular chaperones directly bind to α-synuclein oligomers
- The binding is mediated by hydrophobicity on the oligomer surface
- Bound chaperones significantly attenuate the toxicity of α-synuclein oligomers

Authors

Daniel R. Whiten, Dezerae Cox, Mathew H. Horrocks, ..., Christopher M. Dobson, David Klenerman, Mark R. Wilson

Correspondence

dk10012@cam.ac.uk (D.K.), mrw@uow.edu.au (M.R.W.)

In Brief

Whiten et al. report that the extracellular chaperones clusterin and α_2-macroglobulin bind directly to regions of exposed hydrophobicity on the surface of α-synuclein oligomers. This binding significantly reduces the ability of the oligomers to permeabilize lipid membranes and stimulate the production of reactive oxygen species in a neuronal cell line.

Check for updates

Whiten et al., 2018, Cell Reports *23*, 3492–3500
June 19, 2018 © 2018 The Authors.
https://doi.org/10.1016/j.celrep.2018.05.074

CellPress

OPEN ACCESS
CellPress

Cell Reports
Article

Single-Molecule Characterization of the Interactions between Extracellular Chaperones and Toxic α-Synuclein Oligomers

Daniel R. Whiten,[1,2] Dezerae Cox,[2,4] Mathew H. Horrocks,[1,2,5,6] Christopher G. Taylor,[1] Suman De,[1] Patrick Flagmeier,[1] Laura Tosatto,[1,7] Janet R. Kumita,[1] Heath Ecroyd,[2] Christopher M. Dobson,[1] David Klenerman,[1,3,8,*] and Mark R. Wilson[2,*]
[1]Department of Chemistry, University of Cambridge, Lensfield Road, Cambridge CB2 1EW, UK
[2]Illawarra Health and Medical Research Institute, School of Biological Sciences, University of Wollongong, Wollongong 2522, NSW, Australia
[3]UK Dementia Research Institute, University of Cambridge, Cambridge CB2 0XY, UK
[4]Present address: Department of Biochemistry and Molecular Biology, Bio21 Molecular Science and Biotechnology Institute, University of Melbourne, 30 Flemington Road, Parkville 3052, VIC, Australia
[5]Present address: EaStCHEM School of Chemistry, University of Edinburgh, David Brewster Road, Edinburgh EH9 3FJ, UK
[6]Present address: UK Dementia Research Institute, University of Edinburgh, Edinburgh, UK
[7]Present address: Centre for Integrative Biology, Università degli Studi di Trento, via Sommarive 9, 38123 Trento, Italy
[8]Lead Contact
*Correspondence: dk10012@cam.ac.uk (D.K.), mrw@uow.edu.au (M.R.W.)
https://doi.org/10.1016/j.celrep.2018.05.074

SUMMARY

The aberrant aggregation of α-synuclein is associated with several human diseases, collectively termed the α-synucleinopathies, which includes Parkinson's disease. The progression of these diseases is, in part, mediated by extracellular α-synuclein oligomers that may exert effects through several mechanisms, including prion-like transfer, direct cytotoxicity, and pro-inflammatory actions. In this study, we show that two abundant extracellular chaperones, clusterin and α_2-macroglobulin, directly bind to exposed hydrophobic regions on the surface of α-synuclein oligomers. Using single-molecule fluorescence techniques, we found that clusterin, unlike α_2-macroglobulin, exhibits differential binding to α-synuclein oligomers that may be related to structural differences between two previously described forms of αS oligomers. The binding of both chaperones reduces the ability of the oligomers to permeabilize lipid membranes and prevents an oligomer-induced increase in ROS production in cultured neuronal cells. Taken together, these data suggest a neuroprotective role for extracellular chaperones in suppressing the toxicity associated with α-synuclein oligomers.

INTRODUCTION

The α-synucleinopathies are a group of progressive and, ultimately, fatal neurodegenerative disorders, including Parkinson's disease (PD), dementia with Lewy bodies (DLB) and multiple system atrophy (MSA). The pathological hallmark of these disorders is the selective loss of neurons and the aberrant accumulation of α-synuclein (αS) within protein inclusions in neuronal or glial cells (Chiti and Dobson, 2017). How the aggregation of αS causes disease is still unclear; however, a body of data implicate the direct cytotoxicity of αS oligomers (Chen et al., 2015; Chiti and Dobson, 2017; Ingelsson, 2016; Winner et al., 2011). During the aggregation process, αS oligomers undergo a structural conversion from a relatively unstable species to more stable and compact oligomers that have increased cytotoxicity and resistance to proteinase-K degradation compared to the preceding oligomers (Cremades et al., 2012; Horrocks et al., 2015; Iljina et al., 2016). This conversion occurs before the oligomers are incorporated into fibrillar structures and is a critical step in the aggregation pathway of αS.

αS can account for up to 1% of all cytosolic proteins in neurons, but it is also present in extracellular fluids, including cerebrospinal fluid (CSF) and blood plasma (El-Agnaf et al., 2003). Recent evidence suggests that this extracellular αS significantly contributes to the onset and spreading of disease within the affected brain (Lee et al., 2014). Indeed, as with a growing number of neurodegenerative diseases, it appears that the local spread of pathology may be due to a prion-like propagation process (Aulić et al., 2014; Chiti and Dobson, 2017; Emmanouilidou and Vekrellis, 2016; Marques and Outeiro, 2012). Direct neurotoxicity of extracellular αS has also been observed, which could be caused by the unregulated insertion of αS aggregates into cell membranes and/or neuroinflammatory responses such as microglia activation and generation of intracellular reactive oxygen species (ROS) (Cremades et al., 2012; Fusco et al., 2017; Reynolds et al., 2011; Zhang et al., 2005).

Extracellular chaperones (ECs) are a small class of proteins that act efficiently to enhance the clearance of misfolded proteins from extracellular body fluids. Clusterin (CLU) was the first mammalian EC discovered (Wilson and Easterbrook-Smith, 2000) and has been shown to inhibit the aggregation of a very broad range of proteins, including αS (Yerbury et al., 2007). Thus, since both CLU and aggregates of αS can be present together outside cells, a direct *in vivo* interaction between the

 © 2018 The Authors.
This is an open access article under the CC BY license (http://creativecommons.org/licenses/by/4.0/).

two proteins is feasible and likely. Similarly, another well characterized EC, α_2-macroglobulin (α_2M) (Wyatt et al., 2014), may also interact with αS. A polymorphism in the α_2M gene has been linked with PD, although this link cannot be established for all populations (Krüger et al., 2000; Nicoletti et al., 2002; Tang et al., 2002).

In addition to a possible extracellular interaction, CLU and αS may interact within the cellular environment. Under conditions of endoplasmic reticulum (ER) stress, the secretion of CLU to the extracellular environment is inhibited, and the protein is retrotranslocated from the ER and/or Golgi to the cytosol (Nizard et al., 2007; Zhang et al., 2014). We have recently shown that this process is sufficient to protect cultured neuronal cells and *Drosophila melanogaster* from proteotoxicity associated with the aggregation of the amyotrophic lateral sclerosis (ALS)-linked protein TDP-43 (Gregory et al., 2017). ER stress has been linked to both ALS and PD pathologies; moreover, the overexpression of mutational variants of αS is sufficient to induce ER stress (Gallegos et al., 2015). These observations suggest that a cytosolic interaction between aggregated αS and CLU is also possible. Indeed, CLU has been found colocalized with intracellular αS in patients with a variety of diseases, including cortical Lewy bodies in DLB, brain stem Lewy bodies in PD and DLB, and glial cytosolic inclusions in MSA (Sasaki et al., 2002).

Despite the potentially critical importance of the binding between ECs and αS oligomers, our understanding of the nature of the interaction is limited. Previous work has shown that both CLU and α_2M bind to misfolded proteins to inhibit their aggregation (Wyatt et al., 2013). However, very limited information is available regarding whether specific sizes or structures of oligomers are bound preferentially or on the stoichiometries of binding of chaperone to misfolded client proteins. CLU is known to interact with oligomers of the 40-amino-acid isoform of amyloid-β (Aβ), ranging from dimers up to 50mers (Narayan et al., 2011). CLU also forms stable high-molecular-weight complexes with amorphous aggregates of proteins with a mass ratio of 1:2 (CLU:client) (Wyatt et al., 2009); however, the stoichiometry of complexes formed between either CLU or α_2M and amyloid-forming proteins is not known.

In this report, we used a single-molecule fluorescence technique, termed two-color coincidence detection (TCCD) (Orte et al., 2008a), to show that both CLU and α_2M interact directly with αS oligomers. TCCD allows the properties of two individual proteins, each labeled with one of two spectrally distinct fluorophores, to be studied with high sensitivity (Horrocks et al., 2015). This approach allows the detection of individual species by avoiding measurements of ensemble averages and has been used previously to study the kinetics of αS aggregation (Cremades et al., 2012; Horrocks et al., 2015). In the present study, we demonstrate that the interactions between the chaperones and αS oligomers are inhibited by 4,4′-dianilino-1,1′-binaphthyl-5,5′-disulfonic acid (bisANS), suggesting that the binding involves exposed hydrophobic groups on the surface of the oligomers. Additionally, we show that the chaperones specifically inhibit both an αS-induced increase in lipid membrane permeability and the αS-induced induction of ROS production in neuronal cells.

RESULTS

We first performed TCCD measurements to explore the interaction of the ECs with αS during the aggregation process. To achieve this, we made use of the A90C mutational variant αS, which allows the conjugation of a fluorophore through a single thiol group; previous studies have shown that the conjugation does not significantly change the behavior of the protein from that of wild-type αS (Cremades et al., 2012). αS^{A90C}-AF488 (70 μM) was incubated under aggregation-inducing conditions in the presence of CLU-AF647 (0.7 μM). We took aliquots from the aggregation reaction and monitored, via TCCD, the stoichiometry of any CLU:αS complexes formed. The number of monomers in an oligomer is estimated based on the total fluorescence intensity of the oligomer compared to that of the monomer. Since this is not a direct measurement of the oligomer size, and is approximate, we refer to this as the apparent size. Wherever the term "monomer" is used regarding CLU and α_2M, we are referring to the physiological heterodimer and tetramer, respectively. Under these conditions, both CLU and α_2M were found to greatly inhibit the aggregation of wild-type αS (Figure S1). Previous fluorescence lifetime experiments have shown that AF488 conjugated to αS is not quenched during the oligomerzation of the protein (Cremades et al., 2012). Additionally, since CLU-AF647 did not show any evidence of quenching when bound to unlabeled αS fibrils (Figure S1), we assumed that fluorescence quenching is not a significant factor, particularly when bound to the small oligomers. We were interested to see to which species CLU was bound, and so we searched for coincidence events between the two different fluorophores. After 6 hr of aggregation, we observed coincident events showing that CLU was predominantly bound to small oligomers (approximately tetramers) with an equimolar stoichiometry to αS. For larger oligomers, CLU was found to bind to αS at substoichiometric ratios, with an average CLU/αS ratio of 0.1 calculated for the largest oligomers detected (Figures 1A and 1C). As expected, after 48 hr of aggregation, a larger number of αS oligomers were detected than after 6 hr (Figures 1B and 1D; additional time points are shown in Figure S2). At this later time point, as at 6 hr, a population of larger αS oligomers with a low CLU/αS ratio (approximately 0.06) was detected (Figures 1B and 1D). In addition, a population of "CLU-rich" oligomers were also detected (Figure 1B). These CLU-rich oligomers had an average CLU/αS ratio of 0.8 and generally contained less than 15 αS monomers. Overall, these data show that CLU binds to a wide range of αS species, from at least dimers to oligomers containing 30 αS molecules.

We next studied the formation of α_2M-αS complexes in a manner similar to that used for CLU, as described earlier. αS^{A90C}-AF488 (70 μM) and α_2M-AF647 (0.7 μM) were co-incubated under conditions that facilitated aggregation, and the resulting oligomers were examined using TCCD at various time points. Similarly to CLU, α_2M significantly inhibited the aggregation of αS under these conditions (Figure S1), and again, after 6 hr of incubation, the resulting small αS oligomers showed a broad distribution centered around an equimolar stoichiometry with α_2M (Figures 2A and 2C). The ratio tended to decrease as

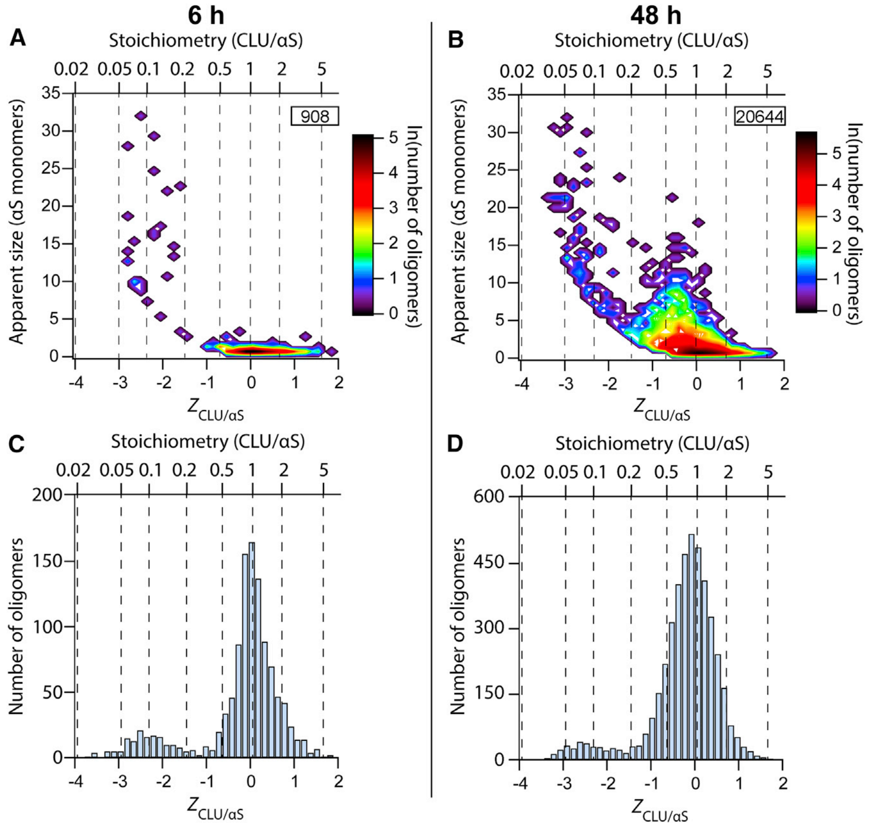

Figure 1. The Ratio of CLU:αS Decreases in Larger Oligomers

αS^{A90C}-AF488 (70 μM) and CLU-AF647 (0.7 μM) were co-incubated in PBS (pH 7.4) at 37°C, with shaking at 200 rpm. The formation of αS-CLU complexes was quantified by single-molecule TCCD.

(A and B) Contour plots of the apparent number of αS monomers constituting an oligomer as a function of the $Z_{CLU/\alpha S}$ value for samples taken from the aggregation reaction after 6 hr (A) and 48 hr (B), respectively. $Z_{CLU/\alpha S}$ represents the logarithm of the apparent ratio of CLU to αS in each oligomer. The data shown are representative of three separate experiments. The numbers in the inserts indicate the number of complexes represented in the plot.

(C and D) Frequency histograms of the number of oligomers at different $Z_{CLU/\alpha S}$ values (for the data shown in A and B, respectively). The dotted lines each indicate a specific CLU: αS^{A90C} stoichiometry (as shown on the upper x axis). The data are representative of three independent experiments.

increasing numbers of αS monomers were present in the oligomer. The largest oligomers detected (consisting of ~30 αS molecules) had an approximate α_2M/αS ratio of 0.03. After αS^{A90C}-AF488 had been incubated with α_2M-AF647 for 48 hr, oligomers were more abundant and tended to contain more α_2M than similarly sized oligomers after 6 hr of incubation (Figures 2B and 2D; additional time points are shown in Figure S2). Small oligomers detected in the aggregation reaction after 48 hr still had an approximate equimolar ratio of α_2M:αS. However, in the largest oligomers detected at 48 hr (containing ~30 αS monomers), the α_2M/αS ratio was around 0.1, approximately three times greater than at the 6-hr time point. In order to further compare the differences in binding stoichiometry at various times during the aggregation reaction, the average numbers of apparent α_2M and αS monomers in different oligomers were plotted (Figures 2E and 2F). This reveals that (1) at all time points, there was a linear dependence of the α_2M:αS ratio on oligomer size (Figure 2E), and (2) the α_2M:αS ratio increased linearly over time for oligomers of all sizes (Figure 2F).

To further investigate the binding of the ECs to the oligomers, we examined whether this binding was a result of hydrophobic interactions by exploiting the effects of the presence of bisANS, a well-established probe of solvent-exposed hydrophobicity (Bothra et al., 1998; Poon et al., 2002; Sheluho and Ackerman, 2001). We first incubated wild-type αS for 9 hr under aggregating conditions and examined the species formed at this time point by super-resolution microscopy using Nile red; we found that they matched the characteristics observed previously for oligomers formed under these conditions (i.e., approximately spherical and <200 nm in diameter; Figure S3). The mixture of monomeric and oligomeric αS was adsorbed to a microplate and next blocked with BSA and treated with a range of concentrations of bisANS to block any exposed hydrophobic regions. Following subsequent incubation with either CLU or α_2M, the extent of chaperone binding was assessed by an ELISA. The bisANS dose-dependently reduced the binding of both CLU and α_2M to the αS oligomers (Figures 3A and 3B), suggesting that the hydrophobic regions exposed on the surface of the oligomers mediates the binding. Neither of the chaperones was found to bind to the BSA blocker or to monomeric αS (Figure S3).

Having shown that the chaperones bind to exposed hydrophobic regions present on αS oligomers, we hypothesized that this binding could act to reduce the cytotoxicity of the oligomers. For this purpose, we first used a methodology that enables the quantification of aggregate-induced toxicity by measuring their effects on the permeability of a lipid bilayer (Flagmeier et al., 2017). In this assay, the Ca^{2+}-sensitive dye Cal-520 is incorporated into surface-tethered vesicles, and Ca^{2+} present in solution can enter the vesicles when the lipid membrane is permeabilized (by, for example, a protein oligomer). The resulting increase in fluorescence intensity can be quantified by total internal reflection fluorescence (TIRF) microscopy and used to determine the extent of membrane permeability. In contrast to a non-EC control protein (BSA), incubation of αS oligomers with both CLU and α_2M significantly reduced the ability of the oligomers to permeabilize the membranes (96 ± 4% and 69 ± 7% protection at a 1:10 substoichiometric ratio of chaperone to monomer, respectively; Figures 3C and 3D). BSA did not reduce the Ca^{2+} influx even when present at an equimolar ratio of BSA:αS (Figure S3). The effect of the

OPEN
ACCESS
CellPress

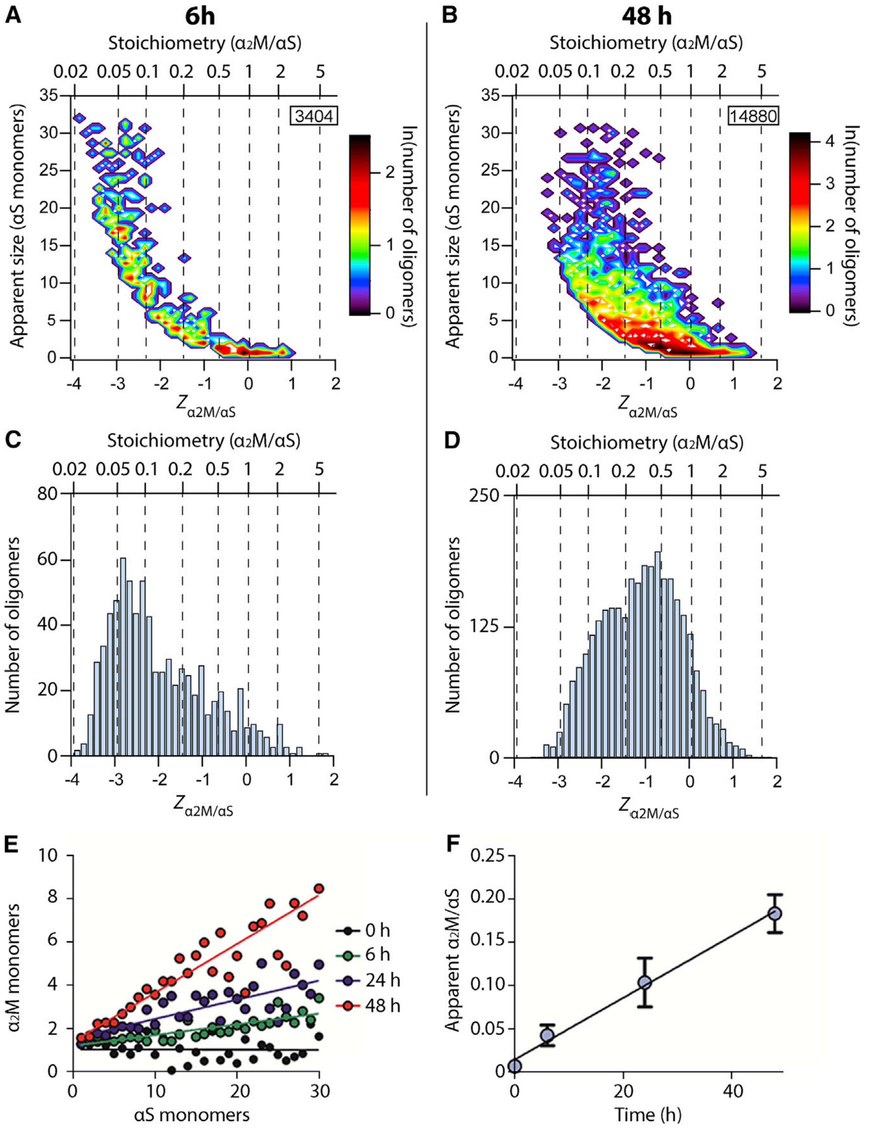

Figure 2. Time-Dependent Changes in the Association of α_2M with αS Oligomers

αS^{A90C}-AF488 (70 μM) and α_2M-AF647 (0.7 μM) were co-incubated in PBS (pH 7.4) at 37°C, with shaking at 200 rpm. The formation of αS-α_2M complexes was quantified by single-molecule TCCD.

(A and B) Contour plots of the apparent number of αS monomers constituting a given oligomer as a function of the $Z_{\alpha2M/\alpha S}$ value for samples taken from the aggregation reaction after 6 hr (A) and 48 hr (B). $Z_{\alpha2M/\alpha S}$ represents the logarithm of the apparent ratio of α_2M to αS in each oligomer. The numbers in the inserts indicate the number of complexes represented in the plot.

(C and D) Frequency histograms of the number of oligomers at different $Z_{\alpha2M/\alpha S}$ values (for the data shown in A and B, respectively). The dotted lines each indicate a specific α_2M:αS^{A90C} stoichiometry (as shown on the upper x axis). Data are representative of three independent experiments.

(E) αS^{A90C}-AF488 (70 μM) and α_2M-AF647 (0.7 μM) were co-incubated in PBS at 37°C, with shaking at 200 rpm, for the indicated time. For each time point, the average numbers of apparent monomers of α_2M and αS per oligomer were calculated. The data were fitted to linear regressions.

(F) The gradients of each linear regression for each time point shown in (A). A time-dependent linear increase in the amount of α_2M found bound to αS can be observed. Data shown are means ± SEM (n = 3).

chaperones was dose dependent, with both chaperones providing >95% protection at a concentration equimolar to that of monomeric αS. Additionally, both CLU and α_2M reduced the αS-induced permeabilization when αS was aggregated in the presence of the chaperone in a 1:100 substoichiometric ratio of chaperone to αS monomer (Figure S3).

To determine whether this effect was sufficient to alter the cellular response to αS oligomers, we measured the effect of both ECs on the cellular production of ROS (Cremades et al., 2012). Intracellular ROS has previously been shown to activate apoptosis in neurons (Jenner, 2003) and is typically one of the first aberrant cellular responses induced by exposure to toxic protein oligomers (Canevari et al., 2004; Zampagni et al., 2011). The oxidation of dihydroethidium (DHE) to ethidium was used to measure the rate of ROS production immediately after the addition of αS oligomers to Neuro-2a cells, in the presence or absence of the ECs. The addition of pre-formed oligomers alone, or of oligomers preincubated with BSA, resulted in an approximately 2.5-fold increase in the rate of ROS production. Preincubation of the oligomers with CLU or α_2M ameliorated their ability to induce ROS formation in cells (Figures 3E and 3F). As neither chaperone alone had any effect on the rate of ROS production, the protective effect is not the result of the chaperones inhibiting the ability of the cells to produce ROS but rather indicates a protective effect conferred by the ECs binding to the oligomers.

DISCUSSION

In the present study, we investigated the direct interaction of ECs with αS under conditions where aggregation occurs. We first used TCCD to examine the stoichiometry of the EC/αS complexes. Using this approach, we have shown that the nature of the binding between the chaperones and αS oligomers is specific for each chaperone and, in the case of CLU, appears to depend on subtle differences in the oligomeric structures. In aliquots taken from aggregation reactions after 6 hr, the stoichiometry of the complexes formed between each EC and αS were similar. Both chaperones tended to bind small oligomers (<5 αS monomers) in an approximate equimolar ratio. Oligomers containing >5 αS monomers were observed to be

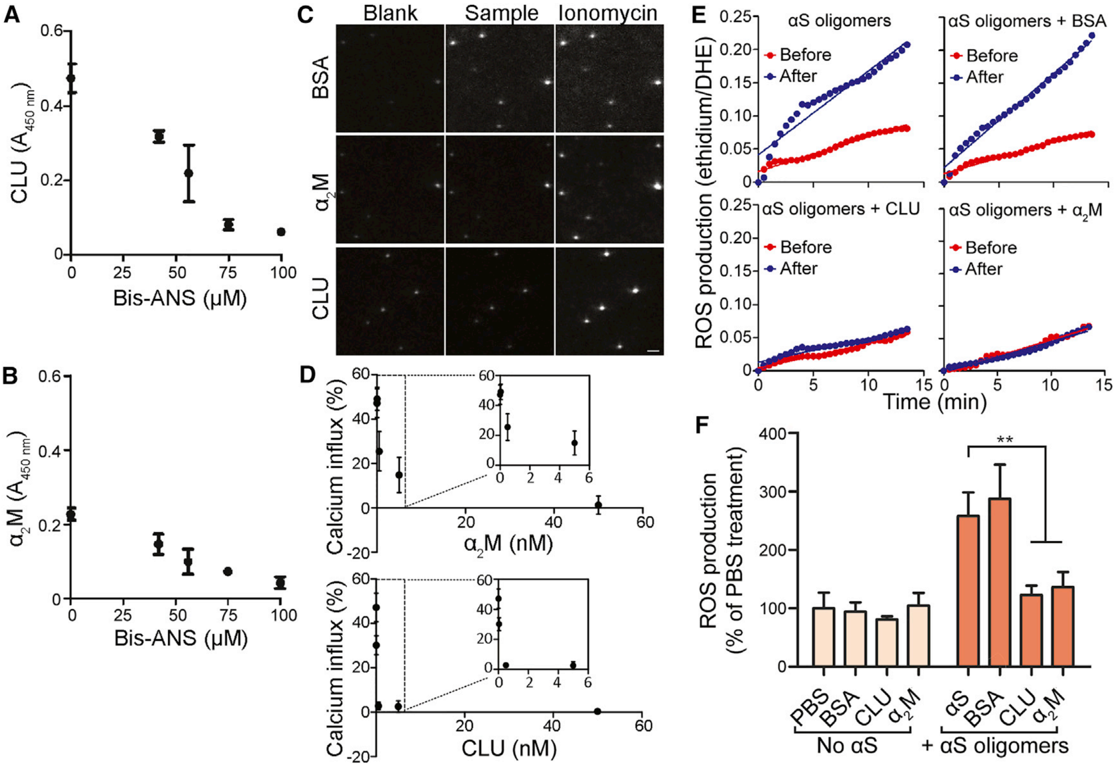

Figure 3. Hydrophobic Shielding Reduces the Ability of αS Oligomers to Induce Membrane Disruption and ROS Production

(A and B) BisANS inhibits the binding of CLU (A) and α_2M (B) to αS oligomers. The chaperones (present at 10 μg • mL^{-1}) were incubated in an ELISA plate pre-coated with aggregated αS, and the amount of bound chaperone was then measured by ELISA. Neither chaperone bound to monomeric αS or the BSA blocker (Figure S3). Data shown are means ± SD of three independent experiments.

(C) αS oligomers were pre-incubated with CLU, α_2M, or BSA and then added to surface-tethered lipid vesicles filled with the Ca^{2+}-sensitive fluorophore Cal-520. The extent of membrane permeabilization was quantified as a percentage of maximum fluorescence produced after incubation with the Ca^{2+} ionophore ionomycin. Example images of vesicles after the addition of the indicated sample are shown. Scale bar (bottom right), 2 μm.

(D) Quantification of the data shown in (C). α_2M or CLU (0.05–50 nM) was incubated with aggregated αS. The extent of membrane permeabilization decreased with increasing chaperone concentration. Data shown are means ± SD of 9 fields of view (at least 800 vesicles) and are representative of two independent experiments.

(E) Aggregated αS preincubated with CLU, α_2M, or BSA (each present at a 1:10 substoichiometric ratio) was added to Neuro-2a (N2a) cells. The rate of ROS production before and after the sample addition was quantified by measuring the oxidation of DHE to ethidium by epifluorescence microscopy. The change in the rate of ROS generation due to the addition of a sample was calculated by subtracting the gradient of the pre-addition line from the gradient of the post-addition line. Example rates of ROS production in a single cell under the indicated conditions are shown.

(F) Quantification of the data shown in (E). The change in the rate of ROS production produced by each sample relative to the buffer-only sample is indicated. The values are means ± SD of approximately 50 cells across three replicate treatments. **$p < 0.01$, analyzed by one-way ANOVA with a Bonferroni post-test.

associated with proportionally less chaperone, suggesting that the number of chaperone-accessible binding sites on the αS oligomer surface does not increase linearly with the number of αS monomers in an oligomer. These data also indicate that, on average, slightly fewer α_2M molecules are associated with the oligomers of all sizes when compared with CLU. This is likely to be the result of the significant difference in size between the two chaperones: α_2M is much larger than CLU (approximately 720 kDa for the α_2M tetramer and 80 kDa for the CLU heterodimer). CLU is known to exist in solution as a polydisperse mixture of oligomers of the heterodimer (Hochgrebe et al., 2000; Poon et al., 2002). It is not known whether these species exhibit variable chaperone activity; however, even a CLU tetramer is approximately half the size of α_2M. As a result, the binding of α_2M to αS is likely to be sterically limited by previously bound chaperones to a greater extent than for CLU. This could potentially help explain the observation that dimeric α_2M is a more efficient chaperone than the native tetramer (Wyatt et al., 2014). The biological function of CLU oligomers is not known, although it has been suggested that they act as reservoirs of a more chaperone-active CLU heterodimer that is released when required (Poon et al., 2002). Unfortunately, the role of these oligomers is difficult to investigate experimentally using single-molecule techniques due to the rapid dissociation of the oligomers upon dilution.

Interestingly, compared to the complexes observed after 6 hr of incubation, a distinct population of CLU/αS complexes emerged at later time points that were relatively large (up to

~15 αS monomers) and CLU rich (CLU/αS ratio = 0.8). One possible explanation for the appearance of this population may be a time-dependent association of additional αS monomers with already formed CLU/αS complexes; however, the clear delineation between this population and those observed at 6 hr suggests that this is not the case. It was recently shown that αS oligomers exhibit a broad distribution of structure-dependent surface hydrophobicity (Bongiovanni et al., 2016). The data presented here indicate that the binding of CLU to the oligomers is mediated by hydrophobicity, suggesting that the ability of CLU to bind to the oligomers is dependent on some aspect of the oligomer structure. Furthermore, the observed time dependence of the differential binding of CLU to αS oligomers cannot be explained by the differential binding of CLU monomers and oligomers, which are both present at the start of the experiment before dilution for single-molecule measurements. This can, however, be explained by CLU binding to αS oligomers of different structures.

Both α_2M and CLU have previously been shown to reduce the toxic effects of Aβ oligomers (Fabrizi et al., 2001; Narayan et al., 2014) and reduce the toxicity of CSF from Alzheimer's disease patients and healthy controls (Drews et al., 2017; Yerbury and Wilson, 2010). This is believed to be a result of the endocytic clearance of the oligomer from the extracellular space through the formation of oligomer-chaperone complexes such as those directly observed in this report. This process is thought to be one of the central systems acting to maintain extracellular proteostasis (Wyatt et al., 2013). Additionally, the work presented here indicates that the binding of ECs to oligomers directly inhibits the latter from aberrantly interacting with lipid membranes. Similarly to other client proteins (Poon et al., 2002), the association between ECs and αS oligomers appears to be mediated by regions of exposed hydrophobicity, evidenced by the inhibition of binding by the hydrophobic probe bisANS. Thus, by interacting with the oligomers, chaperones appear to shield the surface hydrophobicity present on the oligomers. Although the precise mechanism by which αS oligomers confer cytotoxicity is unknown, direct membrane disruption appears to be one of the contributing factors (Poon et al., 2002). Given that hydrophobicity contributes to the interaction between αS and lipid membranes (Pfefferkorn et al., 2012; van Rooijen et al., 2009), it is not surprising that the chaperones inhibited the permeabilization of lipid vesicles caused by αS oligomers. This provides a feasible mechanistic explanation for the observation that the chaperones prevent an increase in ROS production elicited by the oligomers.

Overall, despite evidence that the binding of both α_2M and CLU to αS oligomers is mediated by surface-exposed regions of hydrophobicity on the oligomers, the binding of each chaperone shows unique characteristics. The data suggest that the interaction between the chaperones and αS oligomers appears to depend on both the identity of the chaperone and the structural properties of the oligomer. When considered alongside the chaperone-mediated reduction in αS-induced membrane permeability and ROS production, these data provide evidence to support a neuroprotective role for ECs in the α-synucleinopathies and suggest a mechanism by which these chaperones may operate within other disease contexts.

EXPERIMENTAL PROCEDURES

Protein Purification and Labeling

αS (wild-type and A90C; αS^{A90C}), CLU, and α_2M were purified as described previously (see Cremades et al., 2012; French et al., 2008; and Poon et al., 2002, respectively). αS^{A90C} was labeled with either Alexa Fluor 488 (AF488) C_5 maleimide or Alexa Fluor (AF647) C_2 maleimide (Invitrogen). The αS was first incubated for 15 min with 10 μM DTT at room temperature (RT) to ensure reduction of the engineered cysteine residue. The reduced αS^{A90C} was concentrated to approximately 400 μM using a Vivaspin 500 (10,000 MWCO) and buffer exchanged through a PD-10 column (GE Healthcare Life Sciences) into degassed PBS. The protein was then added to a 1.5-fold molar excess of the functionalized fluorophores, and the tube was flushed with nitrogen to prevent oxidation of the cysteines. The protein was incubated at 4°C overnight, with shaking followed by purification from unreacted fluorophore using a PD-10 column equilibrated in PBS (pH 7.4). The fluorescent labeling of αS^{A90C} has previously been shown to have minimal influence on the aggregation of the protein (Cremades et al., 2012; Horrocks et al., 2015). Similarly, CLU and α_2M (Sigma Aldrich) were individually labeled with N-hydroxysuccinimidyl ester forms of either AF488 or AF647 (Invitrogen). To achieve this, the proteins (each at approximately 2 mg $\cdot$ mL^{-1}) were incubated with a 10-fold molar excess of the functionalized fluorophore for 1 hr at RT or overnight at 4°C. Unconjugated dye was removed by buffer exchange into PBS (or PBS with 0.01% azide in the case of α_2M) using a PD-10 column. The final protein concentration and labeling efficiency were determined according to the manufacturer's instructions.

Aggregation of αS

Any pre-aggregated material present in the monomer stock was first removed from the monomer population by ultra-centrifugation at 90,000 rpm for 1 hr at 4°C. Then, αS was aggregated in the presence or absence of the ECs. When present, the chaperone was used at a molar ratio to αS of 1:100. All aggregations were performed using 70 μM αS in PBS (pH 7.4), with shaking at 200 rpm, 37°C, in an Innova43 Incubator Shaker Series (New Brunswick Scientific). Protein LoBind tubes (Eppendorf) were used to minimize protein adsorption; time point samples were flash frozen in liquid nitrogen for storage before use.

Microfluidics

Single-channel microfluidic devices were used to increase the rate of data acquisition through sample flow and remove the bias for preferentially measuring smaller species that occurs as a result of diffusion. These devices were made of polydimethylsiloxane (PDMS), patterned using a silicon wafer, and bonded to borosilicate glass cover slides by exposure to oxygen plasma. The construction and use of these devices for examining αS aggregation has been described previously (Horrocks et al., 2015).

Single-Molecule TCCD

TCCD measurements were performed with fluorescent proteins present at 50 pM. Dilutions, in freshly filtered (0.02 μm) PBS, were performed immediately before analysis. TCCD measurements were made using a custom-built confocal microscope. Briefly, the intensities of a 488 nm laser (Spectra Physics Cyan, CDRH) and a 633 nm laser (Melles Griot, 25-LHP-151 helium neon [HeNe]) were first attenuated using neutral density filters. The beams were expanded and collimated by passage through a spatial filter (488 nm laser) or telescopic lenses (633 nm laser) before being made concentric with a dichroic mirror (505DRLP Omega Filters). The beams were then directed into the back port of an inverted microscope (Nikon Eclipse TE2000-U) and focused 10 μm into the sample by a Fluor 100X, 1.30 NA, oil-immersion objective (Nikon). The emitted fluorescence was collected by the same objective and passed through a 50 μm pinhole (Melles Griot) before being separated into two channels by a further dichroic mirror (585DRLP, Omega Filters). Emission in each channel was passed through long-pass and band-pass filters (535AF45 and 510ALP Omega Filters for the blue-green channel and 696AF55 and 565ALP Omega Filters for the red channel) and focused on avalanche photodiodes (Perkin-Elmer Optoelectronics, SPCM-14) for quantification.

The apparent size of an oligomer measured by confocal microscopy was calculated by first determining the fluorescence intensity of the monomer.

This was typically given by the average intensity of non-coincident events before the sample was incubated under conditions to promote aggregation. The number of monomers in each coincident burst was then characterized as:

$$N_{Monomers} = \frac{I_D}{I_{MD}} + \frac{I_A}{I_{MA}}$$

where I_D and I_A are the intensities of the coincident burst in the donor and acceptor channels, respectively; and I_{MA} and I_{MD} are the mean monomer intensities in the donor and acceptor channels, respectively (Orte et al., 2008a). In a similar fashion, the natural logarithm of the apparent ratio of chaperone to client in each oligomer (Z) was calculated according to the following equation:

$$Z = \ln\left(\frac{\left(\frac{I_{chaperone}}{Im_{chaperone}}\right)}{\left(\frac{I_{client}}{Im_{client}}\right)}\right)$$

where I refers to the intensity of a peak above the threshold from fluorophores conjugated to the chaperone or client protein, and I_m refers to the intensity of the monomer. These Z values are used to display the ratio of chaperone to client, so that the scale is visually symmetrical around a 1:1 stoichiometry (Z = 0). It should be noted that, in these experiments, the apparent monomer intensity determined from any given dataset is dependent upon the value used to threshold those data. However, since the threshold was determined automatically, as described previously (Clarke et al., 2007; Orte et al., 2008b), the error in the calculated number of monomers labeled with each fluorophore is the same when comparing the two labeled species. Thus, all references to the number of monomers constituting an oligomer refers to apparent monomers, and although the given values scale correctly with each other, they may differ from the true value.

TIRF Microscopy

Borosilicate glass cover slides (24 × 50 mm, thickness number 1; VWR International) for use in TIRF microscopy were cleaned by exposure to oxygen plasma for 30 min (FEMTO plasma system, Diener Electronic). Frame-Seal incubation chambers (Bio-Rad) were attached to the surface of the slides to create wells, which were then coated in aspartic acid (1 mg/mL; Sigma Aldrich) for 15 min. The aspartic acid was removed, and the slide was rinsed with PBS before use. Samples were analyzed at approximately 3 μM monomer equivalents—a concentration that allowed individual aggregates to be resolvable on the surface of the slide. Either 5 μM thioflavin T or 5 nM Nile red was added to the sample before imaging to visualize αS aggregates (Bongiovanni et al., 2016). Measurements were performed on a custom-built inverted optical microscope. The intensities of 405 nm, 532 nm, and 641 nm lasers were attenuated using neutral density filters, after which the beams were circularly polarized using quarter-wave plates specific to each wavelength. The beams were then expanded and collimated using Galilean beam expanders and made concentric using dichroic mirrors before being passed through the back aperture of an inverted microscope and focused using an oil immersion TIRF objective (APON60XO TIRF, Olympus). Fluorescence emission was separated from excitation light using dichroic mirrors (Di02-R532 and Di01-R405/455/561/635 for 532 nm and 405/641 nm excitation, respectively; Semrock) and passed through appropriate filters (BLP01-488R-25, LP02-568RS-25, and BLP01-635R-25 for 405 nm, 532 nm, and 641 nm excitation, respectively; Semrock). The fluorescence was then expanded and focused on an electron-multiplying charge-coupled device (Evolve 512, Photometrics) for imaging.

Super-resolution images were reconstructed using the Drift Calculator and Peak Fit package (GDSC SMLM, University of Sussex) in ImageJ using gain = 37.7 analog to digital units (ADU) per photon, minimum photons >30, and precision <30 nm. Cluster analysis was performed to remove random localizations using DBSCAN (sklearn v0.18.1, Python 2.7); minimum points threshold = 10, and epsilon = 3.

ELISA

αS was aggregated for 9 hr as described earlier, centrifuged at 13,000 × g for 10 min to remove any large aggregates, and then diluted to 2.5 μM. The protein was then adsorbed to a high-binding 96-well microplate (Corning) for 2 hr at RT with gentle shaking. After the incubation, the plate was rinsed with PBS and blocked with 150 μM BSA as described earlier. Some wells were then incubated with bisANS (Sigma) in a concentration range of 0–100 μM to block solvent-exposed hydrophobic regions (Poon et al., 2002). Following this, the wells were incubated with CLU or α_2M for 1 hr at RT (each at 10 μg • mL^{-1} diluted into the BSA blocking solution). The wells were then rinsed five times with PBS before the amount of bound chaperone was quantified using horseradish peroxidase (HRP)/chromogen detection of appropriate antibodies according to the manufacturer's instructions (antibodies and reagents from CLU and α_2M ELISA kits, ab174447 and ab108883, respectively; Abcam).

Single Vesicle Assay

A quantitative vesicle assay was used to measure the ability of αS oligomers to permeabilize membranes as described previously (Flagmeier et al., 2017). αS was aggregated as described earlier, and aliquots were removed and diluted so that the final concentration of αS added to the vesicles was 50 nM. The diluted samples were preincubated for 5 min at RT in the presence or absence of α_2M, BSA, or CLU (concentrations are indicated in the legend for Figure 3) before being added to the solution above POPC lipid vesicles containing Cal-520 (100 μM; Stratech Scientific) tethered to the surface using biotin/neutravidin linkage. A change in the fluorescence as a result of Ca^{2+} (present at 1.3 mM in L-15 buffer; Thermo Fisher Scientific) entering the vesicles was quantified by means of TIRF microscopy using a 488 nm laser for excitation (Toptica Photonics) and emission filters BLP01-488R-25 and FF01-520/44-25 (Semrock). The fluorescence intensity of each vesicle was then normalized to the maximum possible fluorescence intensity of the vesicle measured following incubation with ionomycin (1.4 μM; Sigma). For each sample, the acquisition of 9 fields of view (3 × 3 grid) was automated to prevent user bias.

DHE Assay

DHE was used to measure the intracellular rate of ROS production in Neuro-2a cells, using a method similar to that previously described (Cremades et al., 2012). The cells were cultured in DMEM/Ham's Nutrient Mixture F-12 (Thermo Fisher Scientific) supplemented with 10% (v/v) fetal bovine serum (Bovagen Biologicals) and incubated in a Heracell 150i CO_2 incubator (Thermo Fisher Scientific) under 5% (v/v) CO_2 at 37°C. Cells to be analyzed were seeded in 24-well plates and left to grow to approximately 50% confluency. The cells were rinsed with PBS before DHE (2 μM in PBS) was added. An epifluorescence microscope was used to quantify both the oxidized (ethidium: excitation, 405–435 nm; emission, 440–480 nm) and reduced (DHE: excitation, 502–560 nm; emission, 590–630 nm) forms of DHE. Measurements were taken every 30 s for 15 min before the addition of aggregated αS (30-μM monomer equivalent) with and without preincubation with BSA, CLU, or α_2M (all: 3 μM, 5 min at RT). DHE (2 μM) was present in any sample added to the cells to prevent the dilution of the fluorophore. Measurements were then taken of the same cells for a further 15 min. The ratio of the mean ethidium intensity to the mean DHE intensity before and after the addition of the sample was calculated; a linear regression was fitted to the data, and the gradient of the slope was used to determine the change in the rate of oxidation of DHE within cells. In each experiment, the first two data points collected after the addition of the DHE and sample (i.e., the 0-, 0.5-, 15.5-, and 16-min time points) were excluded from the analysis, as the sample addition briefly disturbed the fluorescence measurement.

SUPPLEMENTAL INFORMATION

Supplemental Information includes three figures and can be found with this article online at https://doi.org/10.1016/j.celrep.2018.05.074.

ACKNOWLEDGMENTS

The authors would like to thank Ms. Beata Blaszczyk and Ms. Swapan Preet for the purification of α-synuclein. D.R.W. and D.C. were supported through an Australian Postgraduate Award. M.R.W. is supported by a Discovery Project grant (DP160100011) from the Australian Research Council (ARC). D.K. is

funded by a European Research Council advanced grant (669237) and the Royal Society. This study is also supported by the Boehringer Ingelheim Fonds (to P.F.), the UK Biotechnology and Biochemical Sciences Research Council (to C.M.D.), the Wellcome Trust (to C.M.D.), and the Cambridge Centre for Misfolding Diseases (to P.F. and C.M.D.). S.D. is grateful for a Marie Curie individual fellowship, and M.H.H. is grateful for a Herchel Smith fellowship and a Junior Research fellowship (Christ's College, Cambridge, UK). L.T. is funded through a Fondazione Umberto Veronesi post-doctoral fellowship 2017.

AUTHOR CONTRIBUTIONS

The study was conceived and designed by M.R.W., D.K., and D.R.W. Experiments were performed by D.R.W., D.C., M.H.H., S.D., P.F., C.G.T., and L.T. Data were analyzed by D.R.W., M.H.H., and S.D. All authors interpreted results, contributed to the manuscript, and approved the final version.

DECLARATION OF INTERESTS

The authors declare no competing interests.

Received: January 10, 2018
Revised: March 26, 2018
Accepted: May 22, 2018
Published: June 19, 2018

REFERENCES

Aulić, S., Le, T.T., Moda, F., Abounit, S., Corvaglia, S., Casalis, L., Gustincich, S., Zurzolo, C., Tagliavini, F., and Legname, G. (2014). Defined α-synuclein prion-like molecular assemblies spreading in cell culture. BMC Neurosci. *15*, 69.

Bongiovanni, M.N., Godet, J., Horrocks, M.H., Tosatto, L., Carr, A.R., Wirthensohn, D.C., Ranasinghe, R.T., Lee, J.-E., Ponjavic, A., Fritz, J.V., et al. (2016). Multi-dimensional super-resolution imaging enables surface hydrophobicity mapping. Nat. Commun. *7*, 13544.

Bothra, A., Bhattacharyya, A., Mukhopadhyay, C., Bhattacharyya, K., and Roy, S. (1998). A fluorescence spectroscopic and molecular dynamics study of bis-ANS/protein interaction. J. Biomol. Struct. Dyn. *15*, 959–966.

Canevari, L., Abramov, A.Y., and Duchen, M.R. (2004). Toxicity of amyloid beta peptide: tales of calcium, mitochondria, and oxidative stress. Neurochem. Res. *29*, 637–650.

Chen, S.W., Drakulic, S., Deas, E., Ouberai, M., Aprile, F.A., Arranz, R., Ness, S., Roodveldt, C., Guilliams, T., De-Genst, E.J., et al. (2015). Structural characterization of toxic oligomers that are kinetically trapped during α-synuclein fibril formation. Proc. Natl. Acad. Sci. USA *112*, E1994–E2003.

Chiti, F., and Dobson, C.M. (2017). Protein misfolding, amyloid formation, and human disease: a summary of progress over the last decade. Annu. Rev. Biochem. *86*, 27–68.

Clarke, R.W., Orte, A., and Klenerman, D. (2007). Optimized threshold selection for single-molecule two-color fluorescence coincidence spectroscopy. Anal. Chem. *79*, 2771–2777.

Cremades, N., Cohen, S.I.A., Deas, E., Abramov, A.Y., Chen, A.Y., Orte, A., Sandal, M., Clarke, R.W., Dunne, P., Aprile, F.A., et al. (2012). Direct observation of the interconversion of normal and toxic forms of α-synuclein. Cell *149*, 1048–1059.

Drews, A., De, S., Flagmeier, P., Wirthensohn, D.C., Chen, W.-H., Whiten, D.R., Rodrigues, M., Vincke, C., Muyldermans, S., Paterson, R.W., et al. (2017). Inhibiting the Ca^{2+} influx induced by human CSF. Cell Rep. *21*, 3310–3316.

El-Agnaf, O.M., Salem, S.A., Paleologou, K.E., Cooper, L.J., Fullwood, N.J., Gibson, M.J., Curran, M.D., Court, J.A., Mann, D.M., Ikeda, S., et al. (2003). Alpha-synuclein implicated in Parkinson's disease is present in extracellular biological fluids, including human plasma. FASEB J. *17*, 1945–1947.

Emmanouilidou, E., and Vekrellis, K. (2016). Exocytosis and spreading of normal and aberrant alpha-synuclein. Brain Pathol. *26*, 398–403.

Fabrizi, C., Businaro, R., Lauro, G.M., and Fumagalli, L. (2001). Role of alpha2-macroglobulin in regulating amyloid beta-protein neurotoxicity: protective or detrimental factor? J. Neurochem. *78*, 406–412.

Flagmeier, P., De, S., Wirthensohn, D.C., Lee, S.F., Vincke, C., Muyldermans, S., Knowles, T.P.J., Gandhi, S., Dobson, C.M., and Klenerman, D. (2017). Ultrasensitive measurement of Ca^{2+} influx into lipid vesicles induced by protein aggregates. Angew. Chem. Int. Ed. Engl. *56*, 7750–7754.

French, K., Yerbury, J.J., and Wilson, M.R. (2008). Protease activation of alpha2-macroglobulin modulates a chaperone-like action with broad specificity. Biochemistry *47*, 1176–1185.

Fusco, G., Chen, S.W., Williamson, P.T.F., Cascella, R., Perni, M., Jarvis, J.A., Cecchi, C., Vendruscolo, M., Chiti, F., Cremades, N., et al. (2017). Structural basis of membrane disruption and cellular toxicity by α-synuclein oligomers. Science *358*, 1440–1443.

Gallegos, S., Pacheco, C., Peters, C., Opazo, C.M., and Aguayo, L.G. (2015). Features of alpha-synuclein that could explain the progression and irreversibility of Parkinson's disease. Front. Neurosci. *9*, 59.

Gregory, J.M., Whiten, D.R., Brown, R.A., Barros, T.P., Kumita, J.R., Yerbury, J.J., Satapathy, S., McDade, K., Smith, C., Luheshi, L.M., et al. (2017). Clusterin protects neurons against cytoplasmic proteotoxicity. Acta Neuropathol. Commun. *5*, 81.

Hochgrebe, T., Pankhurst, G.J., Wilce, J., and Easterbrook-Smith, S.B. (2000). pH-dependent changes in the in vitro ligand-binding properties and structure of human clusterin. Biochemistry *39*, 1411–1419.

Horrocks, M.H., Tosatto, L., Dear, A.J., Garcia, G.A., Iljina, M., Cremades, N., Dalla Serra, M., Knowles, T.P.J., Dobson, C.M., and Klenerman, D. (2015). Fast flow microfluidics and single-molecule fluorescence for the rapid characterization of α-synuclein oligomers. Anal. Chem. *87*, 8818–8826.

Iljina, M., Garcia, G.A., Horrocks, M.H., Tosatto, L., Choi, M.L., Ganzinger, K.A., Abramov, A.Y., Gandhi, S., Wood, N.W., Cremades, N., et al. (2016). Kinetic model of the aggregation of alpha-synuclein provides insights into prion-like spreading. Proc. Natl. Acad. Sci. USA *113*, E1206–E1215.

Ingelsson, M. (2016). Alpha-synuclein oligomers—neurotoxic molecules in Parkinson's disease and other Lewy body disorders. Front. Neurosci. *10*, 408.

Jenner, P. (2003). Oxidative stress in Parkinson's disease. Ann. Neurol. *53* (*Suppl 3*), S26–S36.

Krüger, R., Menezes-Saecker, A.M.V., Schöls, L., Kuhn, W., Müller, T., Woitalla, D., Berg, D., Berger, K., Przuntek, H., Epplen, J.T., and Riess, O. (2000). Genetic analysis of the alpha2-macroglobulin gene in early- and late-onset Parkinson's disease. Neuroreport *11*, 2439–2442.

Lee, H.-J., Bae, E.-J., and Lee, S.-J. (2014). Extracellular α-synuclein—a novel and crucial factor in Lewy body diseases. Nat. Rev. Neurol. *10*, 92–98.

Marques, O., and Outeiro, T.F. (2012). Alpha-synuclein: from secretion to dysfunction and death. Cell Death Dis. *3*, e350.

Narayan, P., Orte, A., Clarke, R.W., Bolognesi, B., Hook, S., Ganzinger, K.A., Meehan, S., Wilson, M.R., Dobson, C.M., and Klenerman, D. (2011). The extracellular chaperone clusterin sequesters oligomeric forms of the amyloid-β(1-40) peptide. Nat. Struct. Mol. Biol. *19*, 79–83.

Narayan, P., Holmström, K.M., Kim, D.-H., Whitcomb, D.J., Wilson, M.R., St George-Hyslop, P., Wood, N.W., Dobson, C.M., Cho, K., Abramov, A.Y., and Klenerman, D. (2014). Rare individual amyloid-β oligomers act on astrocytes to initiate neuronal damage. Biochemistry *53*, 2442–2453.

Nicoletti, G., Annesi, G., Tomaino, C., Spadafora, P., Pasqua, A.A., Annesi, F., Serra, P., Caracciolo, M., Messina, D., Zappia, M., and Quattrone, A. (2002). No evidence of association between the alpha-2 macroglobulin gene and Parkinson's disease in a case-control sample. Neurosci. Lett. *328*, 65–67.

Nizard, P., Tetley, S., Le Dréan, Y., Watrin, T., Le Goff, P., Wilson, M.R., and Michel, D. (2007). Stress-induced retrotranslocation of clusterin/ApoJ into the cytosol. Traffic *8*, 554–565.

Orte, A., Birkett, N.R., Clarke, R.W., Devlin, G.L., Dobson, C.M., and Klenerman, D. (2008a). Direct characterization of amyloidogenic oligomers by single-molecule fluorescence. Proc. Natl. Acad. Sci. USA *105*, 14424–14429.

Orte, A., Clarke, R.W., and Klenerman, D. (2008b). Fluorescence coincidence spectroscopy for single-molecule fluorescence resonance energy-transfer measurements. Anal. Chem. *80*, 8389–8397.

Pfefferkorn, C.M., Jiang, Z., and Lee, J.C. (2012). Biophysics of α-synuclein membrane interactions. Biochim. Biophys. Acta *1818*, 162–171.

Poon, S., Rybchyn, M.S., Easterbrook-Smith, S.B., Carver, J.A., Pankhurst, G.J., and Wilson, M.R. (2002). Mildly acidic pH activates the extracellular molecular chaperone clusterin. J. Biol. Chem. *277*, 39532–39540.

Reynolds, N.P., Soragni, A., Rabe, M., Verdes, D., Liverani, E., Handschin, S., Riek, R., and Seeger, S. (2011). Mechanism of membrane interaction and disruption by α-synuclein. J. Am. Chem. Soc. *133*, 19366–19375.

Sasaki, K., Doh-ura, K., Wakisaka, Y., and Iwaki, T. (2002). Clusterin/apolipoprotein J is associated with cortical Lewy bodies: immunohistochemical study in cases with α-synucleinopathies. Acta Neuropathol. *104*, 225–230.

Sheluho, D., and Ackerman, S.H. (2001). An accessible hydrophobic surface is a key element of the molecular chaperone action of Atp11p. J. Biol. Chem. *276*, 39945–39949.

Tang, G., Zhang, M., Xie, H., Jiang, S., Wang, Z., Xu, L., Hao, Y., Lin, D., Lan, H., Wang, Y., et al. (2002). Alpha-2 macroglobulin I1000 V polymorphism in Chinese sporadic Alzheimer's disease and Parkinson's disease. Neurosci. Lett. *328*, 195–197.

van Rooijen, B.D., Claessens, M.M.A.E., and Subramaniam, V. (2009). Lipid bilayer disruption by oligomeric α-synuclein depends on bilayer charge and accessibility of the hydrophobic core. Biochim. Biophys. Acta *1788*, 1271–1278.

Wilson, M.R., and Easterbrook-Smith, S.B. (2000). Clusterin is a secreted mammalian chaperone. Trends Biochem. Sci. *25*, 95–98.

Winner, B., Jappelli, R., Maji, S.K., Desplats, P.A., Boyer, L., Aigner, S., Hetzer, C., Loher, T., Vilar, M., Campioni, S., et al. (2011). In vivo demonstration that α-synuclein oligomers are toxic. Proc. Natl. Acad. Sci. USA *108*, 4194–4199.

Wyatt, A.R., Yerbury, J.J., and Wilson, M.R. (2009). Structural characterization of clusterin-chaperone client protein complexes. J. Biol. Chem. *284*, 21920–21927.

Wyatt, A.R., Yerbury, J.J., Ecroyd, H., and Wilson, M.R. (2013). Extracellular chaperones and proteostasis. Annu. Rev. Biochem. *82*, 295–322.

Wyatt, A.R., Kumita, J.R., Mifsud, R.W., Gooden, C.A., Wilson, M.R., and Dobson, C.M. (2014). Hypochlorite-induced structural modifications enhance the chaperone activity of human α2-macroglobulin. Proc. Natl. Acad. Sci. USA *111*, E2081–E2090.

Yerbury, J.J., and Wilson, M.R. (2010). Extracellular chaperones modulate the effects of Alzheimer's patient cerebrospinal fluid on Abeta(1-42) toxicity and uptake. Cell Stress Chaperones *15*, 115–121.

Yerbury, J.J., Poon, S., Meehan, S., Thompson, B., Kumita, J.R., Dobson, C.M., and Wilson, M.R. (2007). The extracellular chaperone clusterin influences amyloid formation and toxicity by interacting with prefibrillar structures. FASEB J. *21*, 2312–2322.

Zampagni, M., Cascella, R., Casamenti, F., Grossi, C., Evangelisti, E., Wright, D., Becatti, M., Liguri, G., Mannini, B., Campioni, S., et al. (2011). A comparison of the biochemical modifications caused by toxic and non-toxic protein oligomers in cells. J. Cell. Mol. Med. *15*, 2106–2116.

Zhang, W., Wang, T., Pei, Z., Miller, D.S., Wu, X., Block, M.L., Wilson, B., Zhang, W., Zhou, Y., Hong, J.S., and Zhang, J. (2005). Aggregated alpha-synuclein activates microglia: a process leading to disease progression in Parkinson's disease. FASEB J. *19*, 533–542.

Zhang, F., Kumano, M., Beraldi, E., Fazli, L., Du, C., Moore, S., Sorensen, P., Zoubeidi, A., and Gleave, M.E. (2014). Clusterin facilitates stress-induced lipidation of LC3 and autophagosome biogenesis to enhance cancer cell survival. Nat. Commun. *5*, 5775.

Cell Reports, Volume *23*

Supplemental Information

Single-Molecule Characterization of the Interactions between Extracellular Chaperones and Toxic α-Synuclein Oligomers

Daniel R. Whiten, Dezerae Cox, Mathew H. Horrocks, Christopher G. Taylor, Suman De, Patrick Flagmeier, Laura Tosatto, Janet R. Kumita, Heath Ecroyd, Christopher M. Dobson, David Klenerman, and Mark R. Wilson

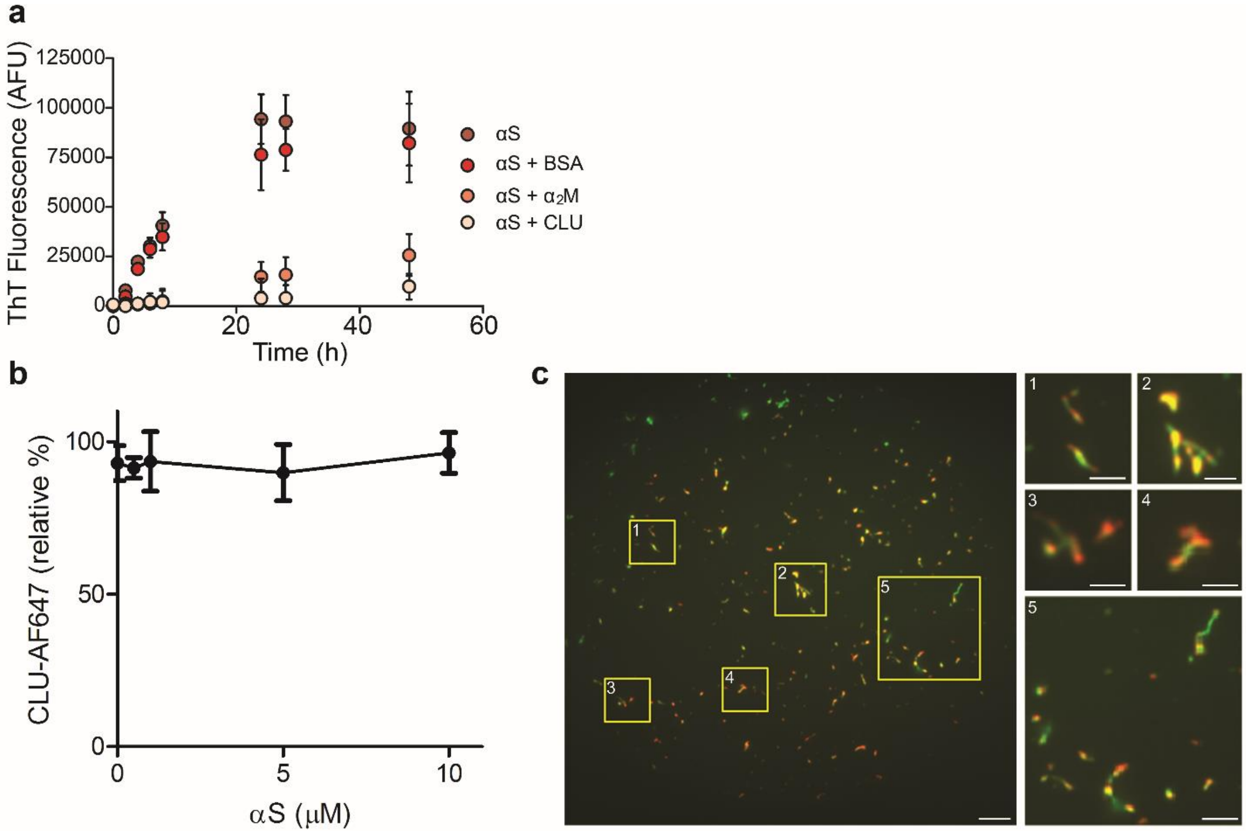

Figure S1. *Related to Figures 1 and 2.* **(a)** αS (70 μM) was incubated alone or with BSA (as a negative control), α_2M or CLU (each at 0.7 μM) for 48 h at 37 °C and 200 rpm shaking**.** The time points were taken during the reaction and the extent of aggregation was quantified by ThT (20 μM) fluorescence using a Clariostar platereader (BMG LabTech; excitation 440-15 nm, emission 500-25 nm). Data shown are means ± SD of three replicate treatments. **(b)** CLU-AF647 (100 nM) was incubated with shaking for 1 h with various concentrations of αS fibrils (equivalent monomer concentrations are shown, diluted from a 70 μM aggregation reaction incubated for 48 h). The absence of significant quenching was observed when the total fluorescence intensity of the solution was measured and found to be independent from the number of fibrils present. **(c)** TIRF microscopy was used to show that CLU can indeed bind to fibrils. Fibrils were identified using thioflavin T and are shown in green, CLU-AF647 is shown in red, coincidence appears yellow. Panels at right show larger versions of areas indicated by numbered yellow boxes. The scale bars are 2 μm for the main panel, and 1 μm for each of the smaller panels.

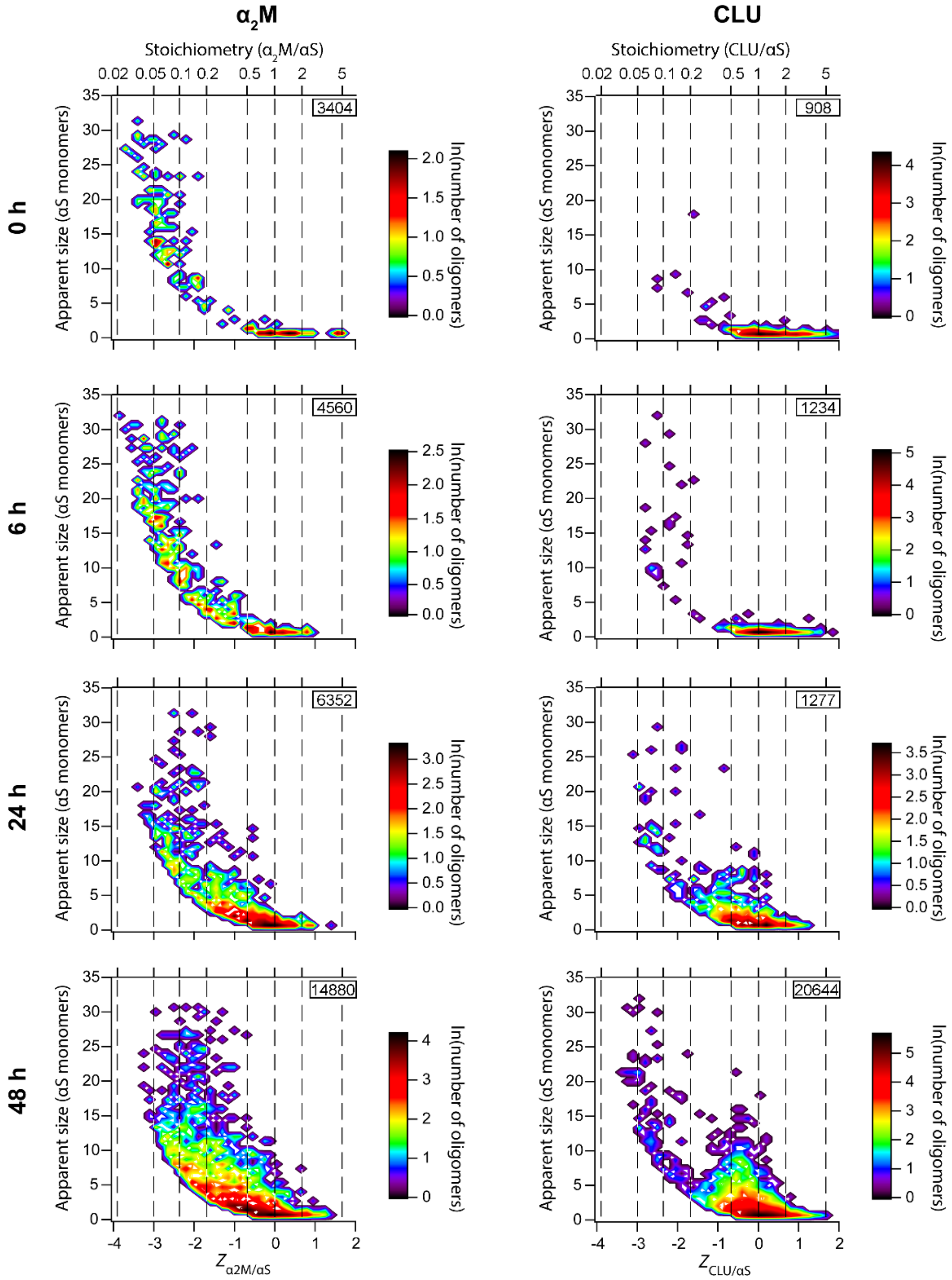

Figure S2. *Related to Figures 1 and 2.* αS^{A90C}-AF488 (70 µM) and either α_2M-AF647 (0.7 µM; left column) or CLU-AF647 (0.7 µM; right column) were co-incubated at 37 °C, with shaking at 200 rpm, in PBS (pH 7.4). Samples were taken from the aggregation reaction at the indicated times and the formation of αS-chaperone complexes was quantified by single-molecule TCCD. $Z_{\text{chaperone}/\alpha S}$ represents the logarithm of the apparent ratio of chaperone to client in each oligomer. The numbers in the inserts indicate the number of complexes represented in the plot. Data shown are representative of at least three separate experiments.

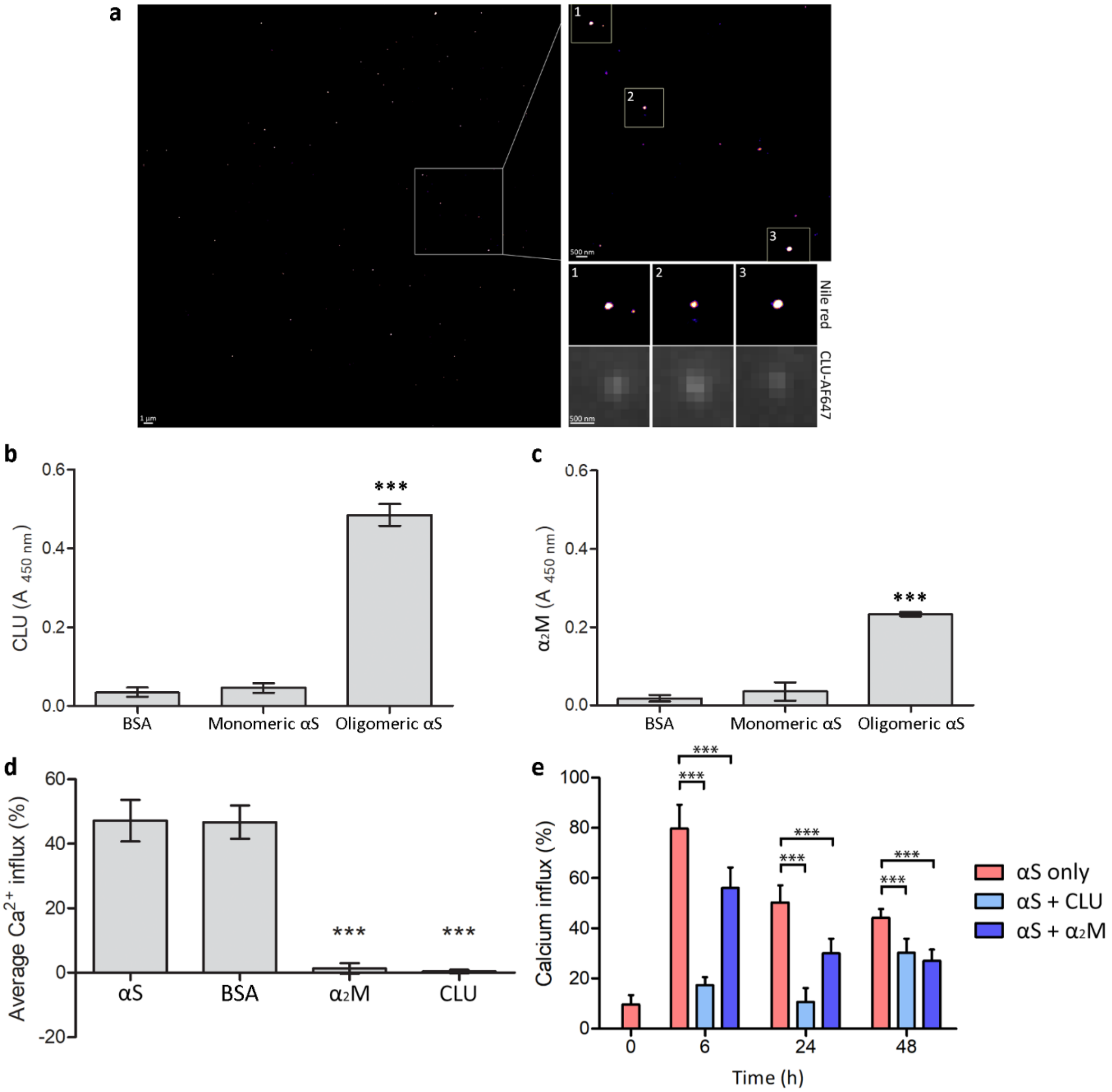

Figure S3. *Related to Figure 3.* **(a)** Monomeric αS was incubated at a concentration of 70 µM under aggregating conditions for 9 h. Aliquots were taken from the aggregation reaction and diluted to a concentration of 3 µM in PBS with 5 nM Nile red for super-resolution imaging. Small aggregates consistent with the expected appearance of oligomers were observed (see the three zoomed panels labelled 'Nile red'). These aggregates were preincubated with CLU-AF647 for 5 min before imaging; panels labelled 'CLU-AF647' are images of the same region as the super-resolution Nile red image but show the diffraction-limited fluorescence of colocalised CLU-AF647. **(b and c)** BSA, monomeric αS or αS incubated under aggregating conditions for 9 h (oligomeric αS) were adsorbed to a microplate. After BSA blocking and incubation with the indicated chaperone the quantity of bound (b) CLU or (c) α_2M was measured by ELISA. *** $p < 0.001$; values shown are means ± SD of three independent experiments. **(d)** αS was incubated under aggregating conditions for 9 h. Aliquots were removed from the aggregation reaction and samples of αS were diluted and added to Cal-520-containing vesicles after preincubation in the presence or absence of BSA, α_2M or CLU at equimolar concentrations to αS (50 nM). Unlike α_2M and CLU, BSA did not counteract the membrane permeability induced by αS. Data shown are means ± SD of 9 fields of view (at least 1000 vesicles); *** $p < 0.001$. **(e)** *CLU and α_2M reduce αS-induced membrane disruption.* α_2M or CLU (700 nM) was incubated with αS (70 µM) for 48 h under aggregating conditions. At the time-points taken both α_2M and CLU decreased the vesicle permeabilisation caused by αS aggregates. Data shown are means ± SD of 16 fields of view (at least 800 vesicles); *** $p < 0.001$.
